# Case report: Unusual development of hepatocellular carcinoma during immunosuppressive treatments against rheumatoid arthritis overlapping Sjögren’s syndrome; cirrhotic steatohepatitis with liver inflammation and fibrosis lurks in autoimmune disorders

**DOI:** 10.3389/fimmu.2023.1089492

**Published:** 2023-02-15

**Authors:** Shuhei Yoshida, Masashi Fujita, Teruhide Ishigame, Yasuyuki Kobayashi, Yuya Sumichika, Kenji Saito, Haruki Matsumoto, Jumpei Temmoku, Yuya Fujita, Naoki Matsuoka, Tomoyuki Asano, Shuzo Sato, Hiroshi Watanabe, Hiroshi Yoshida, Shigeru Marubashi, Yuko Hashimoto, Hiromasa Ohira, Kiyoshi Migita

**Affiliations:** ^1^ Department of Rheumatology, Fukushima Medical University School of Medicine, Fukushima, Japan; ^2^ Department of Gastroenterology, Fukushima Medical University School of Medicine, Fukushima, Japan; ^3^ Department of Hepato-Biliary-Pancreatic and Transplant Surgery, Fukushima Medical University School of Medicine, Fukushima, Japan; ^4^ Department of Diagnostic Pathology, Fukushima Medical University School of Medicine, Fukushima, Japan; ^5^ Department of Internal Medicine, Kita-Fukushima Medical Center, Date, Japan

**Keywords:** methotrexate (MTX), non-alcoholic fatty liver disease (NAFLD), non-alcoholic steatohepatitis (NASH), rheumatoid arthritis (RA), Sjögren’s syndrome (SS), tumor necrosis factor inhibitors (TNFi), hepatocellular carcinoma (HCC)

## Abstract

The sequential progression from chronic liver disease to cirrhosis may be a risk factor for hepatocellular carcinoma (HCC) development. Although HCC originates from hepatitis B virus- or hepatitis C virus-associated liver cirrhosis, it has recently been reported in patients with non-alcoholic steatohepatitis (NASH) with advanced fibrosis. However, little is known about the pathophysiological mechanisms linking HCC to rheumatic disorders, including rheumatoid arthritis (RA). Herein, we describe the case of HCC with NASH complicated by RA and Sjögren’s syndrome (SS). A fifty-two-year-old patient with RA and diabetes was referred to our hospital for further examination of a liver tumor. She received methotrexate (4 mg/week) for 3 years and adalimumab (40 mg/biweekly) for 2 years. On admission, laboratory data showed mild thrombocytopenia and hypoalbuminemia, with normal hepatitis virus markers or liver enzymes. Anti-nuclear antibodies were positive with high titers (x640), and anti-SS-A/Ro (187.0 U/ml; normal range [NR]: ≤6.9 U/mL) and anti-SS-B/La (320 U/ml; NR: ≤6.9 U/mL) antibodies were also high. Abdominal ultrasonography and computed tomography revealed liver cirrhosis and a tumor in the left lobe (S4) of the liver. She was diagnosed with HCC based on imaging findings, and elevated levels of protein induced by vitamin K absence- II (PIVKA-II) were detected. She underwent laparoscopic partial hepatectomy, and histopathological examination revealed steatohepatitis HCC with background liver cirrhosis. The patient was discharged on the 8^th^ day post-operation without any complications. At the 30 months follow-up, no significant evidence of recurrence was observed. Our case suggests that clinical screening for HCC is needed in patients with RA who are at a high risk of NASH, as they may progress to HCC even without elevated liver enzymes.

## 1 Introduction

Non-alcoholic fatty liver disease (NAFLD) is a chronic liver disease that progresses from simple steatosis to nonalcoholic steatohepatitis (NASH) with inflammatory cell infiltration (1). NAFLD can progress to liver cirrhosis and hepatocellular carcinoma (HCC), and several risk factors, including hepatitis virus infection, alcohol abuse, obesity, and diabetes, have been associated with the development of HCC (1–5). Methotrexate (MTX) is currently used as a first-line disease-modifying antirheumatic drug (DMARDs) for treating rheumatoid arthritis (RA) (6, 7). However, persistent transaminitis during low-dose MTX treatment is more likely to occur in patients with RA with NAFLD risk factors such as obesity and type 2 diabetes (8–11). It is also known that long-term administration of methotrexate in patients with RA may lead to liver damage mimicking steatohepatitis-like hepatitis, which can potentially progress to liver cirrhosis (12–14). Nonetheless, the coexistence of HCC and RA has rarely been reported. Sjogren’s syndrome (SS) is an autoimmune disease characterized by lymphocytic infiltration of the salivary and lacrimal glands, clinically manifesting as keratoconjunctivitis sicca and xerostomia (15). Patients with SS have the highest incidence of malignant lymphoproliferative transformation (16, 17). However, an association between SS and HCC has rarely been reported. Here, we present a case of a patient with RA with SS and type 2 diabetes mellitus that presented with liver cirrhosis complicated by HCC. After extensive work-up, including histological evaluation of the liver tissues, a diagnosis of NASH or autoimmune-mediated liver cirrhosis that progressed to HCC was made.

## 2 Case description

**Figure 1:**
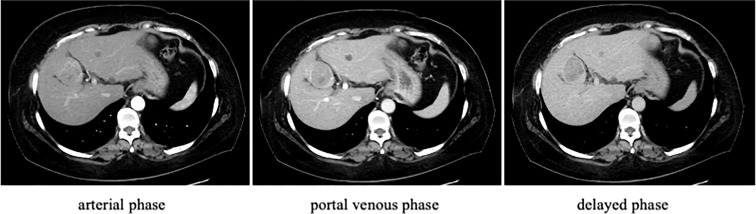
Contrast-enhanced computed tomography (CT) findings on admission. Contrast-enhanced CT of the liver showed a mass in the S4 segment of the liver that darkened in the arterial phase and washed out in the delayed phase. The liver margins were unevenly distributed.

**Figure 2:**
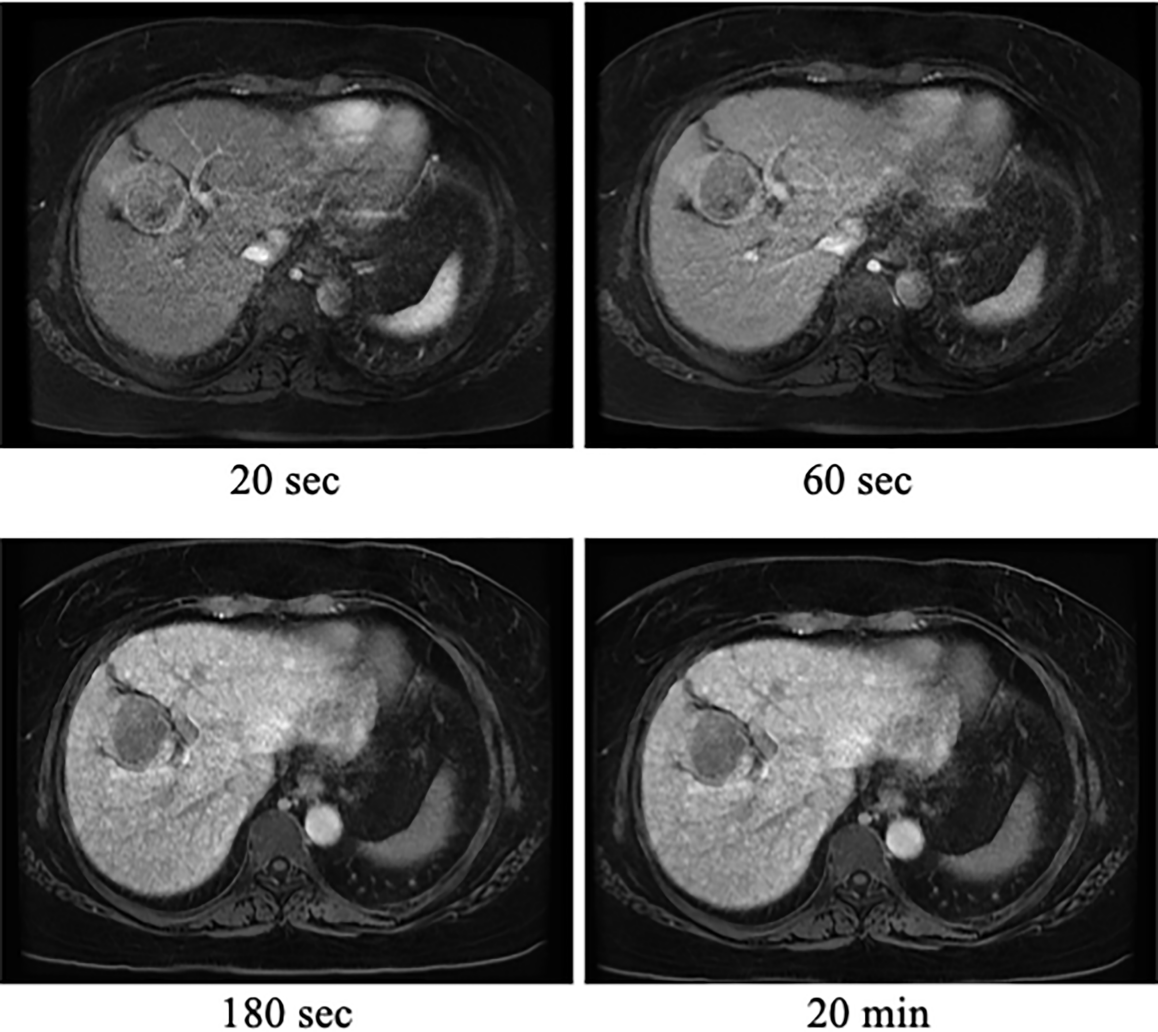
Ethoxybenzyl diethylenetriamine pentaacetic acid (EOB)-enhanced magnetic resonance imaging (MRI) findings. EOB-MRI of the liver revealed liver tumors with dark staining in the early phase (20 s), washout in the delayed phase (180 s), and decreased EOB uptake in the hepatocellular phase (20 min).

**Figure 3:**
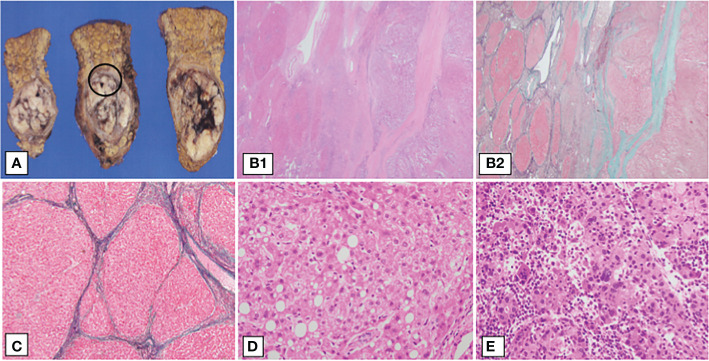
Pathological findings of the patient’s liver resection specimen. Surgical specimens and the pathological findings. **(A)** Cut specimen, **(B1–D)** show magnified images of the black circles. **(B1, B2)** Liver histological findings of hematoxylin-eosin (H.E) staining and Elastica-Masson (E.M) staining, ×40, respectively. Liver fibrosis was observed, indicating F4 cirrhosis. The left half of the specimen is the cirrhotic area and the right half is the hepatocellular carcinoma area. Hepatocellular carcinoma growing in a macrotrabecular pattern or compact pattern. **(C)** Liver histological findings of E.M staining, ×100. Hepatocytes show partial fatty degeneration, and the hepatic lobular structure has disappeared, revealing a fibrous septum. **(D)** Background liver showing fatty degeneration and ballooning of hepatocytes (H&E staining, 400×). **(E)** Histological findings of hepatocellular carcinoma. The main component is poorly differentiated hepatocellular carcinoma. Tumor cells proliferate in a macrotrabecular pattern and show pleomorphism, such as multinucleation and unequal size (H&E staining, 400×).

A 52-year-old Japanese woman with leukopenia and thrombocytopenia was admitted to our hospital. Four years prior to admission, the patient had developed bilateral polyarthritis of the fingers, dryness of the oral cavity, and ocular conjunctiva. Three years prior to admission, she was first diagnosed with type 2 diabetes mellitus, RA, and SS by her previous physician. Blood tests at the initial visit to the previous physician revealed elevated serum levels of C-reactive protein (CRP) (1.75 mg/dL; normal nange [NR]: up to 0.30 mg/dL), anti-cyclic citrullinated peptide (anti-CCP) antibody (13.6 U/mL; NR: up to 4.5 U/mL), anti-nuclear antibody (×640; NR: up to ×40), anti-SS-A/Ro antibody (×16; NR: negative), and anti-SS- B/La antibodies (×8; NR: negative), while platelet (151×10^3^/μL; NR: 150 to 330×10^3^/μL), C3 (80 mg/dL; NR: 73 to 138 mg/dL), C4 (11.6 mg/dL; NR: 11-31 mg/dL) and CH50 (30.5 mg/dL; NR: 25 to 48 mg/dL) were normal. On initial physical examination, the patient had bilateral finger joint swelling and tenderness in the same area, the Disease Activity Score (DAS-28) CRP was 4.2, indicating moderate disease activity of RA. The patient had no history of alcohol consumption or smoking. She had a family history of stomach cancer from her father, but no family history of rheumatic or liver disease. She received alogliptin benzoate (25 mg/day), oral prednisolone (PSL) (5 mg/day) and MTX (4 mg/week) after type 2 diabetes mellitus and RA diagnosis. Despite continuation of oral MTX, RA disease activity remained high. Two years prior to admission, the patient was started on adalimumab (40 mg/biweekly) by subcutaneous injection. After the initiation of adalimumab, the disease was controlled to low disease activity, and serum levels of transaminases remained within normal limits. Four months after starting adalimumab, her physician noted mild leukopenia and thrombocytopenia. Blood tests revealed normal vitamin B12 (393 pg/mL; NR: ≧300) and folic acid (8.9 ng/ml; NR: ≧4), but platelet-associated immunoglobulin G (PA-IgG) levels (162 ng/10^7^ cells; NR: up to 46) were high. She was referred to a hematologist and a bone marrow examination was performed, which showed normal findings. Since the patient had a history of SS and positive PA-IgG, thrombocytopenia was suspected to be either immune thrombocytopenia or drug-induced thrombocytopenia by MTX. One year prior to admission, her leukopenia and thrombocytopenia gradually worsened. A contrast-enhanced CT scan was performed due to a sudden drop in platelet count one month prior to admission, which revealed irregularity of the hepatic margins, development of perigastric collateral vessels, and a 5 cm mass in the S4 of the liver. The mass enhanced in the arterial phase and washed out in the portal venous and delayed phase (**Figure 1**). Portal hypertension due to cirrhosis and HCC was suspected, MTX and adalimumab were immediately discontinued because of liver cirrhosis. The total amounts of drug exposure for MTX and adalimumab were 560 mg and 1680 mg, respectively. The patient was referred to our hospital for further examination. At the time of admission, her tongue was dry and her ocular conjunctiva was hyperemic due to dryness. No lymph nodes were palpable on the body, respiratory sounds were normal, and there were no peripheral sensory abnormalities. The joints were not swollen or tender, and the liver was not palpable; she was obese, with a body mass index of 37.4 kg/m^2^. The blood test results on admission are shown in **Table 1**. Blood tests revealed leukopenia and thrombocytopenia. Hepatitis virus markers were negative and the transaminase level was normal. Antinuclear antibody was positive (640, NR: ≤160). Anti-SS-A/Ro and anti-SS-B/La antibodies were high, at 187 (NR: ≤6.9 U/mL) and >320 U/mL (NR: ≤6.9 U/mL), respectively. Serum cancer markers were elevated with protein induced by vitamin K absence- II (PIVKA-II) at 336 mAU/mL (NR: ≤40 mAU/mL). Total bilirubin was 1.7 mg/dL (NR: 0.4 to 1.5), albumin 3.4 mg/dL (NR: 4.1 to 5.1 mg/dL), and prothrombin time 77.7% (NR: 70 to 130%). A previous contrast-enhanced CT scan showed neither interstitial lung disease nor enlarged lymph nodes throughout the body; No ascites was detected. She was classified as having cirrhosis with a Child-Pugh classification A and a score of 6. The patient’s visual analog scale was 15 and DAS28-CRP was 1.51, so RA was considered as in remission. She met the American-European Consensus Criteria for SS based on her positive anti-SS-A/Ro antibody, positive Schirmer test and positive Saxon test (18). Ethoxybenzyl diethylenetriamine pentaacetic acid-enhanced MRI showed a single mass in the liver with early uptake in the arterial phase, washout patterns at the equilibrium phase and lower signal intensity in the hepatobiliary phase, which strongly suggested HCC (**Figure 2**). A CT scan showed no obvious distant metastases. She was diagnosed with stage II HCC according to the Unio Internationalis Contra Cancrum TNM classification of malignant tumors, 8^th^ edition, and was thus indicated for surgery (19). After admission, the patient was referred to the Department of Hepatobiliary and Pancreatic Surgery. The patient underwent laparoscopic partial hepatectomy. Histopathological images of the specimens are shown in **Figure 3**. The pathological findings were as follows: moderately-to-poorly differentiated HCC; liver (S4) H1; simple nodular type without extranodular growth of up to 38×30×35 mm; eg; fc (+); fc-inf (+); sf (+); s0; vp1; vv0; va0; b0; sm (-); f4 and stage III. The center of the primary hepatocellular carcinoma was necrotic. The background liver had fatty changes and was cirrhotic (F4) according to the new Inuyama classification (20). Additionally, there was an infiltrate of A2-3 lymphocyte-dominated inflammatory cells, mainly in the portal vein area, which was consistent with active chronic hepatitis. No hepatic rosette formation or emperipolesis was observed in the specimens. The tumor cells showed nuclear enlargement, irregular size, and a macrotrabecular pattern, which was more similar to poorly differentiated HCC than to intermediate-differentiated HCC. The margins were negative and the HCC was assessed to be curatively resected. Pathological examination suggested nonalcoholic steatohepatitis and autoimmune-related cirrhosis as the cause of HCC development. She had a score of 13 on the International Autoimmune Hepatitis (AIH) Diagnostic Scoring System (21). She was diagnosed with cirrhosis resulting from a combination of NASH and autoimmune hepatitis, which progressed to HCC. After surgery, MTX was not resumed and the RA remained in remission. Additionally, the HCC did not recur during the 30 months of follow-up period.

**Table 1:** Laboratory findings on admission.

Peripheral blood		Serological tests	
Red blood cells	4.01 × 10^6/^μL	C-reactive protein	0.16 mg/dL (<0.30)
Hemoglobin	13.3 g/dL	Ferritin	111 ng/mL (50–200)
Hematocrit	38.2%	IgG	2923 mg/dL (861–1747)
Platelet	55 × 10^3/^μL	IgA	788 mg/dL (93–393)
White blood cells	1,900/μL	IgM	185 mg/dL (33–183)
Neutrophil	63.0%	C3	75 mg/dL (73–138)
Eosinophil	8.0%	C4	12 mg/dL (11–31)
Monocyte	9.0%	ANA (homogeneous pattern)	1:640 (1:160)
Lymphocyte	20.0%	Anti-ds-DNA Abs	2.9 U/mL (<9.9)
Basophil	0.0%	Anti-Sm Abs	0.6 U/mL (<6.9)
**Blood chemistry**		Anti-SS-A/Ro Abs	187 U/mL (<6.9)
Total protein	8.4 g/dL (6.6–8.1)	Anti-SS-B/La Abs	320 U/mL (<6.9)
Total bilirubin	1.7 mg/dL (0.4–1.5)	Hyaluronic acid	200.8ng/mL (<50)
Albumin	3.4 g/dL (4.1–5.1)	Collagen type IV	8.3 ng/mL (<4.4)
Aspartate aminotransferase	29 IU/L (13–30)	Anti-mitochondrial Abs	(-)
Alanine aminotransferase	18 IU/L (10–42)	Anti-mitochondrial M2 Abs	1.6 U/mL (<6.9)
Lactate dehydrogenase	235 IU/L (124–222)	Anti-centromere Abs	< 5.0 U/mL (<10)
Γ-Glutamyl transpeptidase	25 IU/L (13–64)	Anti-LKM-1 Abs	<5 Index (<17)
Alkaline phosphatase	237 IU/L (106–322)	Mac-2 binding protein glycosylated isomers	4.57 COI (<1.0)
Creatine kinase	46 U/L (59–248)	α-fetoprotein	6.2 ng/mL (<8.8)
Blood urea nitrogen	9 mg/dL (8–20)	CEA	2.6 ng/mL (<5.0)
Creatinine	0.48 mg/dL (0.65–1.07)	CA 19-9	4.8 U/mL (<37.0)
Na	141 mEq/L (138–145)	PIVKA-II	336 mAU/mL (<40)
K	3.7 mEq/L (3.6–4.8)	**Coagulation tests**	
Cl	108 mEq/L (101–108)	Prothrombin time	77.7% (70-130)
Glucose	95 mg/dL (73–109)	activated partial thromboplastin time	36.1 seconds (26.9-38.1)
Hemoglobin A1c	5.0% (4.9–6.0)	**Microbiological tests**	
TSH	1.16 μIU/mL (0.5–5)	HBs Ag	(-)
Free T3	2.78 pg/mL (2.3–4)	Anti-HCV Ab	(-)
Free T4	1.08 ng/dL (0.9–1.7)	HIV-Ab	(-)
		Nontreponemal test	(-)

TSH, thyroid stimulating hormone; Ig, immunoglobulin; ANA, antinuclear antibodies; Abs, antibodies; Anti-ds-DNA, anti-double stranded-DNA; Anti-Sm, anti-smith; Anti-LKM-1, anti-liver-kidney-microsome type 1; CEA, carcinoembryonic antigen, CA 19-9, carbohydrate antigen 19-9; PIVKA II, protein induced by vitamin K absence-II; HBs Ag, hepatitis B virus surface antigen; Anti-HCV Ab, anti-hepatitis C virus antibody; HIV-Ab, human immunodeficiency virus antibody.

## 3 Discussion

HCC often develops because of chronic hepatitis or cirrhosis (22). Herein, we report a case of HCC during immunosuppressive therapy for RA and discuss its possible etiology. Our patient was presumed to have sustained chronic liver damage caused by steatohepatitis or autoimmune-mediated hepatic inflammation due to the coexistence of RA, SS, and diabetes. In addition, long-term use of MTX may result in the progression of liver damage and fibrosis (12–14). Histologically, MTX hepatotoxicity includes macrovascular steatosis, ballooning degeneration, and fibrosis, which are also characteristics of NASH (12). Although the cumulative dose of MTX was relatively low in this case, there is a possibility that MTX-related liver damage could explain the clinical course, including the development of liver cirrhosis. In this case, liver histology revealed interface hepatitis with mononuclear cell infiltration, steatosis, and pericellular/bridging fibrosis, which were also accompanied by hepatocyte ballooning, demonstrating the concurrence with the histological features of steatohepatitis and immune-mediated liver injury. It was difficult to clinically differentiate between NASH and autoimmune-mediated liver damage in the histology of the resected liver tissues adjacent to HCC in this case. However, we decided that NASH-related liver damage could be the major cause of pre-existing liver cirrhosis since AIH overlap was not justified according to the international AIH diagnostic scoring system (AIH score, 13) (21). In contrast to NASH, most other liver diseases can be diagnosed based on clinical features and serological tests (23). In our practice, it should be recognized that the pathological features of NASH or autoimmune-related steatohepatitis liver damage can occur in other forms of clinically defined liver disease in patients with RA.

Epidemiological data suggest that HCC attributed to viral infection is declining, while cases of NASH-related HCC are significantly increasing (24–26). Although RA has been linked to several malignancies, a population-based cohort study has demonstrated that RA is associated with a reduced risk of developing HCC and cirrhosis-associated complications (27). Inflammatory cytokines, including tumor necrosis factor-α and interleukin-6, and their downstream targets, nuclear factor kappa B, c-Jun N-terminal kinase, and signal transducer and activator of transcription 3, may drive inflammation-associated HCC (28). NAFLD, and type 2 diabetes mellitus lead to the release of multiple pro-inflammatory cytokines, including tumor necrosis factor-alpha and interleukin-6, which favor the development of hepatic steatosis and inflammation within the liver, preceding HCC development (29–31). The estimated prevalence of liver fibrosis was shown to be approximately 5%, with NAFLD reaching 30% in SS patients and being positively associated with body mass index (32). Despite the absence of extraglandular manifestations of SS, these combined risk factors may have contributed to the development of steatohepatitis in this case. Although NAFLD is a multifactorial disease with metabolic disorders, immune cell- mediated inflammatory processes are thought to be involved in the progression to NASH and its transition to HCC (33). Autoimmunity may have some pathogenetic roles in the inflammatory processes of NASH (34). Indeed Forty-eight percent (26/54) of NASH cases were positive for anti-nuclear (ANA) and shared the histological findings suggesting autoimmune hepatitis (34). Therefore, it is possible that the coexistence of autoimmune diseases, RA and SS, may have some roles in the development of cirrhotic steatohepatitis in this case. Taken together the present case report suggests that the induction proinflammatory cytokines under the inflammatory processes of RA with NASH can be important mechanisms of liver inflammation, fibrosis, and carcinogenesis. The liver is an immunological organ composed with innate immune cells with a high exposure to circulating antigens (35). In NASH, liver steatosis triggers immune-cell activation (36). These hepatic immune cell landscapes may potentially contribute to the development of steatohepatitis. Although treatment with tumor necrosis factor inhibitors (TNFi) is not associated with increased risk of overall cancer, some prospective studies demonstrated a small increased risk of lymphoma and squamous cell cancer in patients with RA treated with TNFi (37); therefore, the immunosuppressive effects of TNFi might be related to the progression of HCC since this case had been treated with adalimumab for a limited period. During treatment with TNFi, close monitoring of the tumor and progression to HCC should be considered, especially for patients at high risk of HCC in patients with liver cirrhosis. Additional case reports are warranted to validate our observations and clarify the corresponding immunopathogenic mechanisms for HCC progression. Nevertheless, further investigation is necessary to elucidate the factors associated with HCC development in patients with RA and NAFLD of various grades of fibrosis.

Persistent transaminitis during low-dose MTX treatment is more likely to occur in patients with RA with NAFLD risk factors (8–11, 38). However, persistent transaminitis cannot be detected in patients with steatohepatitis who develop advanced liver cirrhosis (39). MTX treatment can be related to the development of persistent transaminitis in patients with RA and NAFLD risk factors, and it is also necessary to perform a prospective follow-up for liver fibrosis including Fib-4 index or platelet counts in patients with RA with normal levels of transaminases (8–11). This case report suggests the need to monitor liver fibrosis in patients with RA with NAFLD risk factors during low-dose MTX treatment. Unfortunately, the accurate diagnostic imaging of focal hepatic lesions and the measurements of tumor markers (AFP and PIVKA-II) for HCC were insufficient for early diagnosis in this case. Physicians should remind that regular survey using imaging of the liver or tumor markers are crucial to early diagnose cirrhotic steatohepatitis and HCC in patients with RA with risk factors, such as obesity, DM and the history of MTX use.

In conclusion, obesity and type 2 diabetes mellitus are the most important predictive factors for NASH- or NASH-mediated liver fibrosis during RA treatment. The progression of underlying NASH to HCC seems to occur in the processes of hepatic inflammation and fibrosis in patients with RA, particularly in those receiving MTX. Quantitative evaluation of liver fibrosis, in addition to liver injury, is useful for identifying patients with RA who are at high risk for developing liver cirrhosis and HCC.

## Data availability statement

The original contributions presented in the study are included in the article/supplementary material. Further inquiries can be directed to the corresponding author.

## Ethics statement

Ethical review and approval was not required for the study on human participants in accordance with the local legislation and institutional requirements. The patients/participants provided their written informed consent to participate in this study. Written informed consent was obtained from the individual(s) for the publication of any potentially identifiable images or data included in this article.

## Author contributions

SY, HY, KM were involved with the conception of the work. MF, YS, KS, HM, JT, YF, NM, TA, SS, HW, HY and HO contributed to the treatment and collection of data. YK and YH performed histopathological evaluation of the liver and tumors. TI performed the patient’s surgery. SY, KM wrote the first draft of the manuscript. All authors contributed to the article and approved the submitted version.
